# Mesenteric and omental lymphatic malformations in children: seven-year surgical experience from two centers in China

**DOI:** 10.1186/s12887-024-04808-w

**Published:** 2024-05-23

**Authors:** Jiayu Yan, Yao Fu, Shuting Liu, Yuzuo Bai, Yajun Chen

**Affiliations:** 1 Department of General Surgery, Beijing Children’s Hospital, Capital Medical University, National Center for Children’s Health, Beijing, 100045 China; 2 School of Public Health, China Medical University, Shenyang, 110122 China; 3 Department of Pediatric Surgery, Shengjing Hospital of China Medical University, Shenyang, 110004 China

**Keywords:** Children, Mesenteric lymphatic malformation, Omental lymphatic malformation, Surgical treatment

## Abstract

**Purpose:**

To compare the clinical characteristics, surgical management and prognosis of mesenteric lymphatic malformations (ML) and omental lymphatic malformations (OL) in children.

**Methods:**

This retrospective study included 148 ML patients and 53 OL patients who underwent surgical treatment at two centers between January 2016 and December 2022. Details about the patients’ clinical characteristics, cyst characteristics, preoperative complications, surgical methods, and prognosis were retrieved and compared.

**Results:**

No significant differences in sex ratio, prenatal diagnosis, or age of diagnosis were noted between ML and OL patients. Vomiting was more common in ML patients than in OL patients (46.6% vs. 22.6%, *P* = 0.002), but OL patients were more likely to be misdiagnosed (35.8% vs. 18.9%, *P* = 0.012). The size of the cysts in OL patients was significantly larger than that in ML patients (14.0 [4.0–30.0] vs. 10.0 [2.0–50.0] cm, *P*<0.001), and cysts with turbid fluid were more common in OL patients (38.0% vs. 20.6%, *P*<0.001). More OL patients than ML patients had preoperative hemorrhage or infection of cysts (41.5% vs. 31.8%, *P*<0.016). Cyst excision was performed in 137 (92.6%) ML patients and 51 (96.2%) OL patients, and the incidence of postoperative complications was lower (12.6% vs. 4.2%, *P* = 0.165) among OL patients. The main postoperative complications included adhesive ileus and recurrence of cysts. Additionally, more OL patients than ML patients were treated with laparoscopic surgery (69.8% vs. 39.2%, *P*<0.001).

**Conclusions:**

There were differences in clinical characteristics, cyst characteristics and preoperative complications between ML and OL patients. Cyst excision was the most common surgical method that was used to treat both ML and OL patients, and laparoscopic surgery could be a feasible surgical approach for treating OL patients with a good prognosis.

**Trial registration:**

Retrospectively registered.

## Background

Abdominal lymphatic malformations are rare, accounting for only 5% of lymphatic malformations, but they are relatively common in the mesentery and omentum [1–3]. The reported incidence of mesenteric lymphatic malformations (ML) and omental lymphatic malformations (OL) is approximately 1/250,000–1/20,000 [3–5]. Considering that ML cysts are often inseparable from the mesentery and intestinal wall, surgical treatment may include ML excision with segmental bowel resection, so a multidisciplinary approach, including medications, sclerotherapy and surgical treatment, is increasingly recommended for the treatment of ML to achieve better results [6–8]. In contrast, OL cysts are less likely to adhere to neighboring viscera and are mainly found in the greater omentum [9, 10]. The recommended treatment for OL is complete surgical resection. The initial symptoms of ML and OL patients are usually vague and almost similar, but symptoms can be aggravated when the size of a cyst increases or complications occur [11, 12]. In addition, apart from the absence of intermixing between the fluid and loops of the bowel being a possible clue to the presence of OL, preoperative imaging examinations can hardly distinguish OL from ML [13]. Therefore, surgery is still the mainstream treatment for abdominal lymphatic malformations, with the aim of relieving obvious symptoms and locating the lymphatic malformations.

Laparoscopic surgery, including robotic-assisted and laparoscopic-assisted surgery, has become an option for treating ML and OL, but previous reports are sparse with small sample sizes [14–16]. In addition, to our knowledge, no studies have systematically compared the clinical characteristics, surgical management and prognosis of ML and OL. Therefore, to provide clinicians with a detailed understanding of these two diseases, we reported experience of treating ML and OL with surgery, including open surgery and laparoscopic surgery, at two tertiary university hospitals in China.

## Methods

### Patients

After being approved by the Ethics Committee of Beijing Children’s Hospital (Approval number: [2023]-E-088-R), we conducted a retrospective, observational, multicenter study of ML and OL patients who were treated at Beijing Children’s Hospital, Children’s National Medical Center, China (Center 1), and Shengjing Hospital of China Medical University (Center 2), from January 2016 to December 2022. Eligible participants were identified by reviewing their electronic medical records, and all the patients who were included in this study underwent surgical treatment and were confirmed to have ML or OL by postoperative histopathology.

### Study design

Clinical data of patients that needed to be collected were discussed and determined before the study began. Jiayu Yan and Shuting Liu collected clinical data, including inpatient, outpatient, and follow-up data, from their hospitals. The inpatient data included clinical characteristics (sex, prenatal diagnosis, age of diagnosis, presenting symptoms, misdiagnosis before admission, laboratory examinations, and preoperative imaging examinations), preoperative complications, surgical details, and pathological data. According to previous studies, the preoperative complications of ML and OL mainly included hemorrhage, infection and rupture of the cyst, as well as intestinal or omental volvulus caused by the cyst [6]. However, hemorrhage and infection of cysts are often concomitant and challenging to distinguish [11].

The surgical details and pathological data were extracted from surgical records and postoperative pathological results to summarize the cyst characteristics and surgical methods. The cyst characteristics included the specific location, classification, pathological type, cyst size, and cyst content. The specific locations of ML varied depending on the mesentery involved, and included intestinal ML, colonic ML and combined ML; the specific locations of OL varied depending on the omentum involved, and included greater ML and lesser ML [6]. Considering that the previously reported classifications of ML could not be applied to OL, a simple classification of OL reported was used in this study, including Group 1 (patients with clinical features due to the size of the cyst), Group 2 (patients with clinical features due to the preoperative complications of the cyst), and Group 3 (patients without clinical features but whose cysts were incidentally detected) [4, 6, 17]. According to the number and size of the cysts contained, the pathological types included macrocystic-type (single or multilocular, ≥ 1 cm), microcystic-type (single or multilocular, < 1 cm) and mixed cystic-type (multilocular) [18, 19]. Both ML and OL are almost cystic lymphatic malformations, making it impossible to measure their specific length, width and height; thus, in our study, the size of the cysts refers to the length of the entire cyst that was obtained from surgical records for patients undergoing open surgery and from preoperative imaging findings for patients undergoing laparoscopic surgery. The ML and OL cysts could contain serous, chylous, or turbid fluid, including serosanguinous fluid caused by hemorrhage or infection of the cyst [6, 11]. The choice of surgical approach for all the included patients depended on the surgeon’s preference, and surgical approaches included open surgery (including laparoscopic conversion to open surgery) and laparoscopic surgery (including laparoscopic-assisted surgery and robot-assisted surgery).

The outpatient data mainly included postoperative ultrasound results. Before June 2023, Jiayu Yan and Shuting Liu conducted telephone follow-up interviews with patients who were enrolled at their respective hospitals. The telephone interviews collected information about postoperative ultrasound results at other hospitals, postoperative complications and their managements. Postoperative complications mainly included recurrence (including residual cysts) and adhesive ileus, and these complications were identified based on hospitalization data, postoperative ultrasound results, and telephone interviews.

For analysis, we compared the clinical characteristics of ML and OL in children and analyzed the prognosis of various surgical methods. In addition, we also tried to identify the factors that influence the choice of open surgery or laparoscopic surgery for treating ML and OL patients.

### Statistical analysis

Statistical analysis was performed using IBM SPSS Statistics for Windows, Version 26.0, IBM Corporation. Variable normality was assessed with Kolmogorov-Smirnov or Shapiro-Wilk tests with additional visual inspection of the Q-Q plots. Continuous variables with a normal distribution are presented as the means ± standard deviations and were analyzed with Student’s t test. Continuous variables with a nonnormal distribution are presented as the median and range, and were analyzed with the Mann-Whitney test. Categorical variables are expressed as numbers (percentages) and were analyzed with the χ2 test or Fisher’s exact test. Multivariate analysis was performed with binary logistic regression using the Wald test. *P* < 0.05 (2-sided) was considered statistically significant.

## Results

### Patient characteristics

**Fig. 1:**
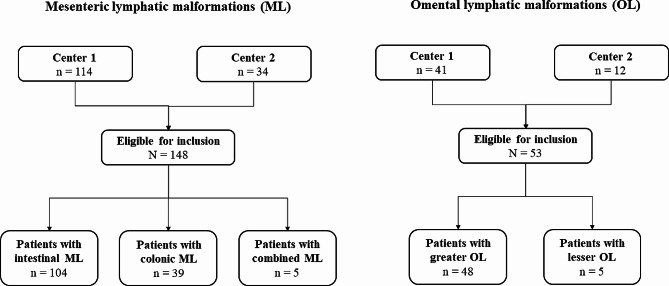
Study patient selection flowchart. Center 1: Beijing Children’s Hospital, Children’s National Medical Center, China; Center 2: Shengjing Hospital of China Medical University

A total of 148 ML patients and 53 OL patients were enrolled in this study from the two centers during the study period (Fig. 1). According to the surgical records, 104 ML (104/148, 70.3%) were located at the intestinal mesentery, 39 (39/148, 26.3%) ML were located in the mesocolon, and 5 (5/148, 3.4%) ML involved both the intestinal mesentery and mesocolon. Among the OL patients, 48 (48/53, 90.6%) OL were in the greater omentum, and 5 (5/53, 9.4%) OL were in the lesser omentum.

**Fig. 2:**
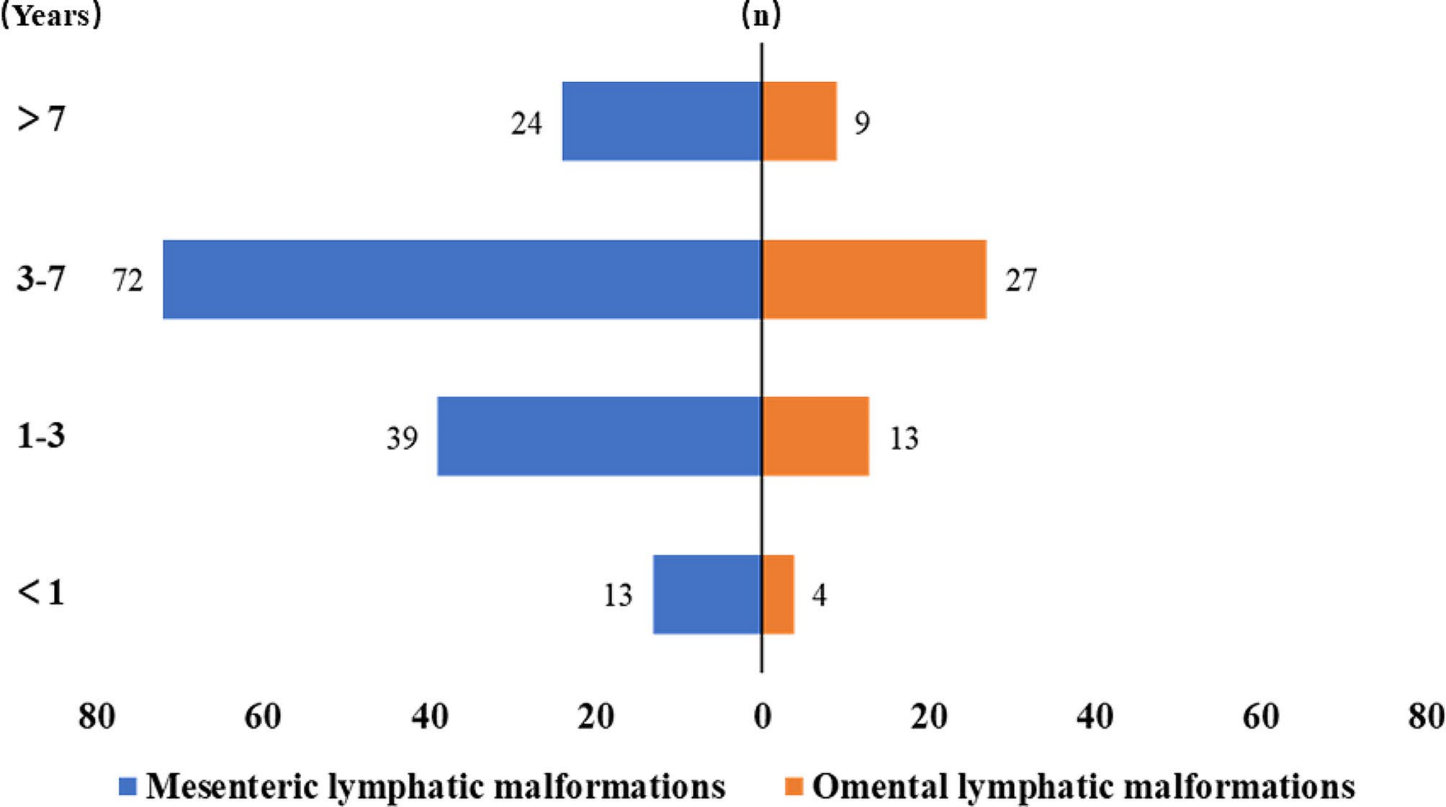
Distribution of ML and OL patients by age

**Fig. 3:**
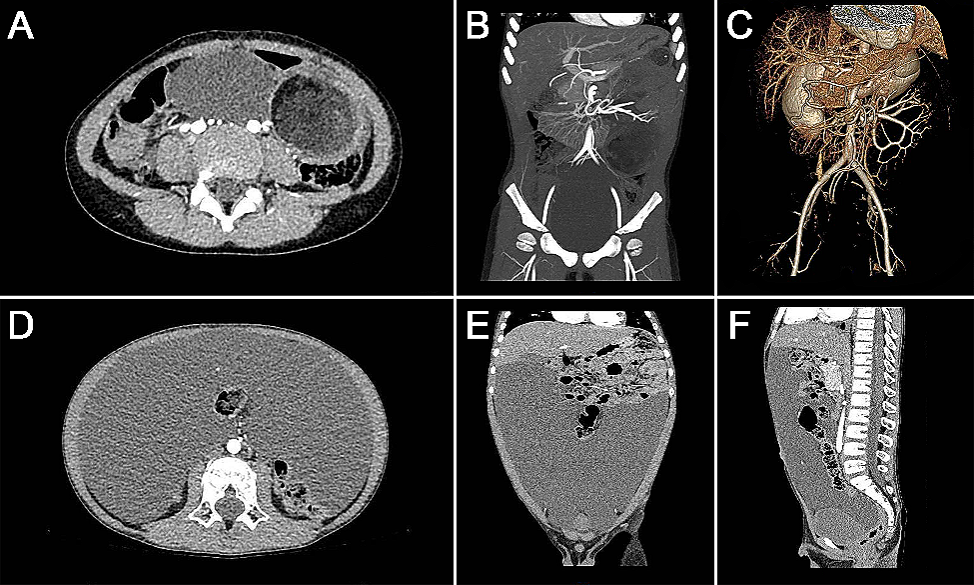
Typical images of ML and OL on abdominal CT. (**A**-**C**) Typical images of ML on abdominal CT: **A**, axial CT image, a mixed cystic mass with wall thickening in the left abdomen; **B**, CT image reconstruction, the blood supply of the mass was the superior mesenteric artery; **C**, computed tomography angiography, the superior mesenteric vessels were rotating 360° counterclockwise. (**D**-**F**) Typical images of OL on abdominal CT: **D**, axial CT image, a large amount of low-attenuation intra-abdominal fluid; **E**, coronal CT image, abdominal mass effect as well as upward bowel displacement; **F**, sagittal CT image, abdominal mass effect as well as backward bowel displacement

Table 1 shows the clinical characteristics of the ML and OL patients. No significant differences between the ML and OL patients were noted in terms of sex ratio, prenatal diagnosis, or age of diagnosis. Peritoneal effusion or an abdominal cystic mass was found in 8 patients (8/201, 4.0%) by prenatal ultrasound at gestational ages ranging from 26 to 39 weeks. The median ages of diagnosis in ML and OL patients was 3.8 [0.1–14.1] years and 3.8 [0.0-16.8] years, respectively, and nearly half of the ML (72/148, 48.6%) and OL (27/53, 50.9%) patients were aged 3–7 years at diagnosis (Fig. 2). Abdominal pain was the predominant clinical symptom in both ML and OL patients (70.9% vs. 60.4%, *P* = 0.156), but vomiting was more common in ML patients than in OL patients (46.6% vs. 22.6%, *P* = 0.002). OL patients were more likely to be misdiagnosed than ML patients (35.8% vs. 18.9%, *P* = 0.012). Gastroenteritis (10 cases in the ML patients and 5 in the OL patients) and peritoneal effusion (3 cases in the ML patients and 4 in the OL patients) were the main causes of suspicion before the definite diagnosis. In addition, the laboratory results at admission showed that OL patients had a higher percentage of infection (45.3% vs. 29.7%, *P* = 0.040). Abdominal ultrasound (95.3% vs. 96.2%, *P*>0.999) and CT (81.1% vs. 77.4%, *P* = 0.560) were the most common preoperative imaging examinations that were used in both the ML and OL patients (Fig. 3).

**Table 1:** Clinical characteristics of patients with ML and OL

Characteristics	ML (*n* = 148)	OL (*n* = 53)	*P* value^**^
Sex, n (%)			0.311
Male	90 (60.8)	28 (52.8)	
Female	58 (39.2)	25 (47.2)	
Prenatal diagnosis, n (%)	5 (3.4)	3 (5.7)	0.437
Age (years)	3.8 [0.1–14.1]	3.8 [0.0-16.8]	0.598
Symptoms, n (%)			
Incidental diagnosis	9 (6.1)	7 (13.2)	0.100
Abdominal pain	105 (70.9)	32 (60.4)	0.156
Vomiting	69 (46.6)	12 (22.6)	0.002
Abdominal distention	50 (33.8)	11 (20.8)	0.077
Abdominal mass	32 (21.6)	7 (13.2)	0.184
Fever	35 (23.6)	15 (28.3)	0.501
Diarrhea	14 (9.5)	3 (5.7)	0.567
Misdiagnosis before admission, n (%)	28 (18.9)	19 (35.8)	0.012
Laboratory examination, n (%)			0.040
Normal	104 (70.3)	29 (54.7)	
Infection^*^	44 (29.7)	24 (45.3)	
Imaging examinations, n (%)			
Ultrasound	141 (95.3)	51 (96.2)	>0.999
CT	120 (81.1)	41 (77.4)	0.560
MRI	6 (4.1)	1 (1.9)	0.678

ML: Mesenteric lymphatic malformations; OL: Omental lymphatic malformations

^*^The laboratory data collected included C-reactive protein and white blood cells. The patients with C-reactive protein > 8 mg/L or white blood cells > 10 × 10^9^/L were considered to be infected

^**^Mann-Whitney test or χ2 test

### Cyst characteristics and preoperative complications

Regarding cyst characteristics, there were significant differences between ML and OL patients in the cysts’ pathological types, sizes and contents (Table 2). Macrocystic-type cyst was more common in OL patients (94.3% vs. 78.4%, *P* = 0.047). The size of the cysts in OL patients was significantly larger than that in ML patients (14.0 [4.0–30.0] vs. 10.0 [2.0–50.0] cm, *P*<0.001). In addition, cysts with chylous fluid and turbid fluid were more common in ML patients (33.1% vs. 4.0%, *P*<0.001) and OL patients (38.0% vs. 20.6%, *P*<0.001), respectively. However, there was no significant difference in classification between ML and OL patients.

**Table 2:** Comparison of cyst characteristics and preoperative complications between patients with ML and OL

Variable	ML (*n* = 148)	OL (*n* = 53)	*P* value^***^
Classifications, n (%)^*^			0.233
Group 1	70 (47.3)	25 (47.2)	
Group 2	69 (46.6)	21 (39.6)	
Group 3	9 (6.1)	7 (13.2)	
Pathological types, n (%)			0.047
Macrocystic-type	116 (78.4)	50 (94.3)	
Microcystic-type	8 (5.4)	3 (5.7)	
Mixed cystic-type	24 (16.2)	0 (0.0)	
Cyst size (cm)	10.0 [2.0–50.0]	14.0 [4.0–30.0]	<0.001
Cyst contents, n (%)	136 (12 N/A)	50 (3 N/A)	<0.001
Serous	63 (46.3)	29 (58.0)	
Chylous	45 (33.1)	2 (4.0)	
Turbid	28 (20.6)	19 (38.0)	
Preoperative complications, n (%)			
Hemorrhage or infection	47 (31.8)	22 (41.5)	0.016
Volvulus^**^	29 (19.6)	3 (5.7)	0.199
Rupture	2 (1.4)	2 (3.8)	0.284

ML: Mesenteric lymphatic malformations; OL: Omental lymphatic malformations

^*^Group 1: patients with clinical features due to the size of the cyst, Group 2: patients with clinical features due to the preoperative complications of the cyst, Group 3: patients without clinical features but incidentally detected

^**^Volvulus referred to intestinal volvulus in patients with ML and omental volvulus in patients with OL

^***^Mann-Whitney test or χ2 test

Hemorrhage or infection was the predominant preoperative complication in both ML and OL patients, but the incidence differed significantly. More OL patients than ML patients had preoperative hemorrhage or infection of cyst (41.5% vs. 31.8%, *P*<0.016).

### Surgical methods and outcomes

**Fig. 4:**
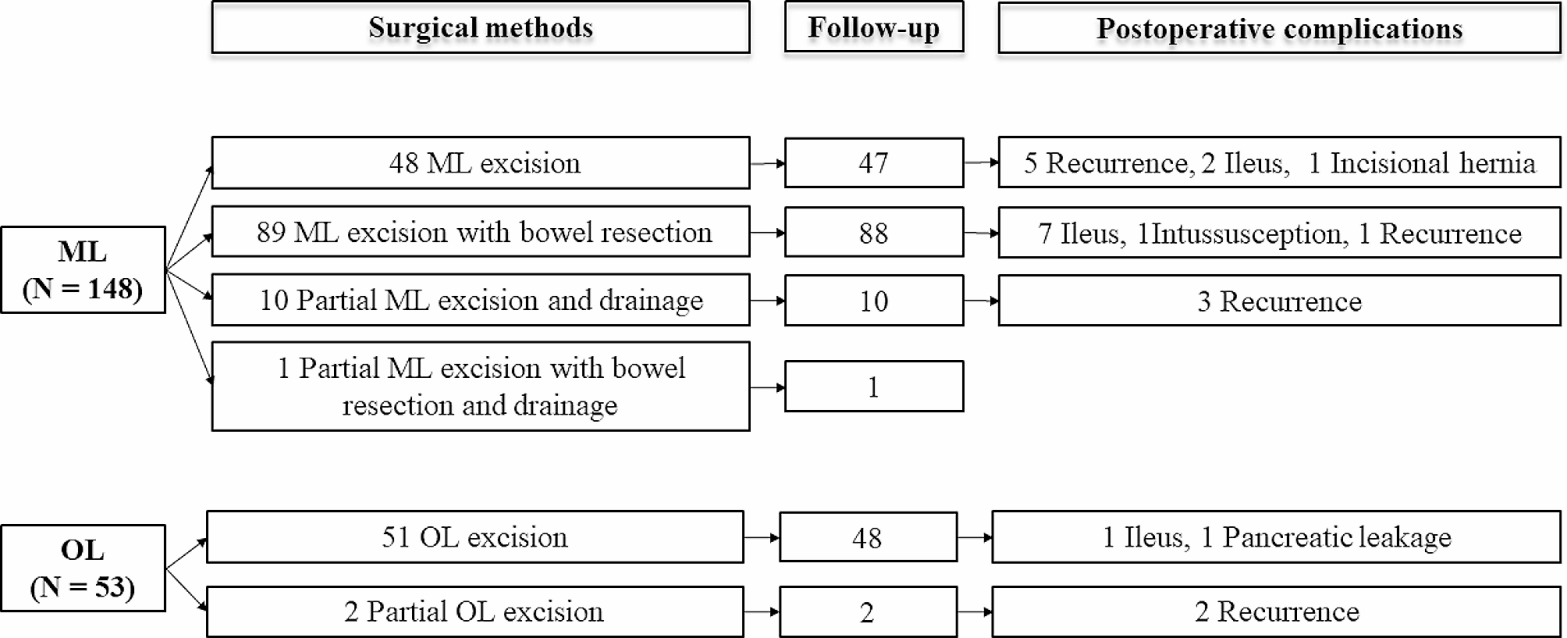
Surgical methods and postoperative complications of ML and OL patients

Regarding the specific surgical methods, 48 (48/148, 32.4%), 89 (89/148, 60.1%), 10 (10/148, 6.8%) and 1 (1/148, 0.7%) ML patients underwent ML excision, ML excision with segmental bowel resection, partial ML excision and drainage, and partial ML excision with segmental bowel resection and drainage, respectively (Fig. 4). Of the OL patients, 51 (51/51, 96.2%) and 2 (2/53, 3.8%) underwent OL excision and partial OL excision, respectively. In total, complete cyst excision was performed in 137 (92.6%) ML patients and 51 (96.2%) OL patients. Among these patients, up to our follow-up, the incidence of postoperative complications in OL patients was lower (4.2% vs. 12.6%, *P* = 0.165). Recurrence of cysts (11/196, 5.6%) and adhesive ileus (10/196, 5.1%) were the main postoperative complications in all 196 children (146 ML patients and 50 OL patients).

As shown in Tables 3 and 4, more OL patients than ML patients underwent laparoscopic surgery (69.8% vs. 39.2%, *P*<0.001). Among ML patients, those who underwent laparoscopic surgery had a smaller average cyst size (8.0 [3.0–16.0] vs. 10.0 [2.0–50.0] cm, *P* = 0.001) and a greater proportion of cysts with serous fluid (61.5% vs. 36.9%, *P* = 0.014). Further multivariable analysis revealed that smaller cysts influence the choice of laparoscopic surgery for ML patients (OR, 1.1; 95% CI, 1.0-1.2; *P* = 0.006). However, no factors that might influence the choice of laparoscopic surgery for OL patients were identified.

**Table 3:** Factors influencing the choice of laparoscopic or open surgery for ML

Characteristics	Open surgery (*n* = 90)	laparoscopic surgery (*n* = 58)^*^	*P* value^**^	Logistic regression, *P* value^***^	OR, 95% CI
Sex, n (%)			0.661	——	——
Male	56 (62.2)	34 (58.6)			
Female	34 (37.8)	24 (41.4)			
Age, years, n (%)	3.6 [0.1–14.1]	4.0 [0.2–11.0]	0.376	——	——
≤ 3	36 (40.0)	16 (27.6)	0.233	——	——
3–7	39 (43.3)	33 (56.9)			
>7	15 (16.7)	9 (15.5)			
Symptoms, n (%)					
Incidental diagnosis	5 (5.6)	4 (6.9)	0.738	——	——
Abdominal pain	66 (73.3)	39 (67.2)	0.426	——	——
Vomiting	39 (43.3)	30 (51.7)	0.318	——	——
Abdominal distention	35 (38.9)	15 (25.9)	0.102	——	——
Abdominal mass	18 (20.0)	14 (24.1)	0.551	——	——
Fever	23 (25.6)	12 (20.7)	0.496	——	——
Diarrhea	8 (8.9)	6 (10.3)	0.768	——	——
Cyst size, cm, n (%)	10.0 [2.0–50.0] 86 (4 N/A)	8.0 [3.0–16.0] 57 (1 N/A)	0.001	0.006	1.1 (1.0-1.2)
≤ 5	13 (15.1)	17 (29.8)	0.055	——	——
5–10	41 (47.7)	27 (47.4)			
>10	32 (37.2)	13 (22.8)			
Location, n (%)			0.994	——	——
Small intestine	63 (70.0)	41 (70.7)			
Colon	24 (26.7)	15 (25.9)			
Combined	3 (3.3)	2 (3.4)			
Cyst contents, n (%)	84 (6 N/A)	52 (6 N/A)	0.014	0.070	——
Serous	31 (36.9)	32 (61.5)			
Chylous	31 (36.9)	14 (26.9)			
Turbid	22 (26.2)	6 (11.5)			
Preoperative complications, n (%)					
Hemorrhage or infection	29 (32.2)	18 (31.0)	0.880	——	——
Volvulus	20 (22.2)	9 (15.5)	0.316	——	
Rupture	1 (0.9)	1 (1.9)	>0.999	——	

ML: Mesenteric lymphatic malformations

^*^One patient underwent robot-assisted surgery

^**^Mann-Whitney test or χ2 test

^***^Wald test

**Table 4:** Factors influencing the choice of laparoscopic or open surgery for OL

Characteristics	Open surgery (*n* = 16)	laparoscopic surgery (*n* = 37)	*P* value^*^
Sex, n (%)			0.354
Male	10 (62.5)	18 (48.6)	
Female	6 (37.5)	19 (51.4)	
Age, years, n (%)	3.4 [0.0-9.6]	4.5 [0.6–16.8]	0.146
≤ 3	6 (37.5)	11 (29.7)	0.785
3–7	8 (50.0)	19 (51.4)	
>7	2 (12.5)	7 (18.9)	
Symptoms, n (%)			
Incidental diagnosis	3 (18.8)	4 (10.8)	0.419
Abdominal pain	10 (62.5)	22 (59.5)	0.835
Vomiting	4 (25.0)	8 (21.6)	>0.999
Abdominal distention	3 (18.8)	8 (21.6)	>0.999
Abdominal mass	3 (18.8)	4 (10.8)	0.419
Fever	4 (25.0)	11 (29.7)	>0.999
Diarrhea	0 (0.0)	3 (8.1)	0.545
Cyst size, cm, n (%)	15.0 [5.0–30.0] 15 (1 N/A)	11.0 [4.0–23.0] 35 (2 N/A)	0.081
≤ 5	1 (6.7)	6 (17.1)	0.149
5–10	2 (13.3)	11 (31.4)	
>10	12 (80.0)	18 (51.4)	
Location, n (%)			0.632
Greater omentum	14 (87.5)	34 (91.9)	
Lesser omentum	2 (12.5)	3 (8.1)	
Cyst contents, n (%)	16	34 (3 N/A)	0.864
Serous	9 (56.3)	20 (58.8)	
Chylous	1 (6.3)	1 (2.9)	
Turbid	6 (37.5)	13 (38.2)	
Preoperative complications, n (%)			
Hemorrhage or infection	8 (50.0)	14 (37.8)	0.409
Volvulus	2 (12.5)	1 (2.7)	0.213
Rupture	0 (0.0)	2 (5.4)	>0.999

OL: Omental lymphatic malformations

^*^Mann-Whitney test, χ2 test or Fisher’s exact test

## Discussion

In the present study, we first systematically compared the clinical characteristics and prognosis of ML and OL patients with the largest sample sizes from two centers, and we found that there were differences in clinical characteristics, cyst characteristics and preoperative complications between ML and OL patients. OL patients were more prone to misdiagnosis and infection before they were definitely diagnosed. Correspondingly, compared with that in ML patients, cyst size in OL patients was significantly larger, and cysts with turbid fluid were more common in OL patients. In addition, our findings demonstrated that cyst excision was the most common surgical method that was used to treat both ML and OL patients, and laparoscopic surgery could be a feasible surgical approach for OL patients with a good prognosis.

A total of 148 ML patients and 53 OL patients were evaluated, and both groups exhibited a predominance of males, similar to previous studies [3–5]. However, with the development of prenatal ultrasound, it was possible to detect excessive peritoneal effusion or abdominal cystic masses in fetuses during pregnancy, and it seemed impossible to distinguish ML from OL [20, 21]. Before the cysts developed complications, most ML and OL patients were asymptomatic or had mild symptoms and only received conservative treatments without imaging examinations. These conditions were often misdiagnosed, sometimes as gastroenteritis [4]. In addition, even after the occurrence of complications, the symptoms of both diseases vary, are nonspecific and difficult to diagnose and distinguish [5]. As observed in our study, except for vomiting, the presenting symptoms of ML and OL patients did not differ significantly. Vomiting often indicates bowel obstruction, which might be related to compression of the intestine by the enlarging cyst or the occurrence of intestinal volvulus [22, 23]. Our previous studies confirmed a high rate of intestinal volvulus in ML patients [6, 11]. Compared with OL patients, ML patients might have more urgent symptoms, so it is recommended that imaging examinations be performed as soon as possible. Consequently, the ML patients in our study had a lower misdiagnosis rate.

Interestingly, the laboratory results showed that OL patients had a higher percentage of infection. There are two possible reasons for this phenomenon. First, as observed in our study, more OL patients had hemorrhage of cysts at admission, and hemorrhage could activate the body’s immune system, leading to the mild elevation of inflammatory markers, such as C-reactive protein [24, 25]. Second, cysts with severe hemorrhage might be more susceptible to infection. In adults with intracerebral hemorrhage, the size of the hematoma was a predictor of infection development [26]. In ML patients, the size of the cyst has been shown to indicate the presence of preoperative hemorrhage or infection. Our results also showed that OL patients had significantly larger cysts than ML patients, and cysts with turbid fluid were more common in OL patients. However, it was difficult to accurately distinguish between hemorrhage and infection from the cysts’ appearance, so the infection process after hemorrhage could not be further analyzed. In addition, in our study, C-reactive protein > 8 mg/L or white blood cells counts > 10 × 10^9^/L indicated an infection according to the reference ranges of Beijing Children’s Hospital, possibly leading to some bias in the results.

Previous studies have shown that complete surgical resection is an effective and durable treatment for abdominal lymphatic malformations with a good prognosis [2, 27, 28]. However, for some complex abdominal lymphatic malformations with multicentric and diffusely infiltrated lymphatic malformations involving important organs, such as type III and type IV ML reported by Kim SH et al., complete surgical resection is very difficult and has a high recurrence rate; therefore, medications and sclerotherapy could be the first choice of treatment. However, the long-term prognosis still needs further study [6, 28]. For OL patients, the recommended treatment is complete surgical resection. Since it is challenging to distinguish ML and OL through preoperative imaging examinations, surgery is necessary to treat patients suspected of having the two diseases mentioned above [2]. Our study revealed that OL patients had a better surgical prognosis than ML patients, especially those with complete OL excision, none of whom experienced the recurrence of cysts. This may be related to the high proportion (94.3%) of macrocystic-type cysts in OL patients. During the surgical treatment of OL patients with macrocystic-type cysts, the involved omentum could be removed to reduce recurrence. In the surgical treatment of ML, however, surgeons should avoid bowel resection whenever possible, and some small cysts in ML patients with microcystic-type or mixed cystic-type cysts cannot be identified by the naked eye [9, 28]. Therefore, ML patients are more likely to experience a recurrence after surgical treatment.

Finally, we observed that, compared with ML patients, more OL patients underwent laparoscopic surgery without any definite influencing factors. This finding suggests that, consistent with previous studies, almost all OL patients were eligible for laparoscopic surgery [10, 15, 29]. Previously reported laparoscopic surgeries for abdominal lymphatic malformations include two surgical approaches. One is complete laparoscopic surgery, which is widely used in adult patients with a large abdominal cavity, and the other is laparoscopic-assisted exploration to first locate and decompress the cyst of lymphatic malformations, followed by cyst excision through a slightly expanded umbilical incision, which is more commonly used in pediatric patients [16, 30–32]. Large cysts in pediatric patients could impair surgical visualization during laparoscopic surgery. Therefore, according to our study, in the surgical treatment of OL, surgeons’ experience might be the main factor influencing the specific surgical approach that is chosen.

There are some significant limitations to the interpretation of our results. First, due to the retrospective nature of our study, the cyst characteristics of each patient, especially cyst classification and cyst size, cannot be guaranteed to be entirely correct, as they are summarized from surgical records and preoperative imaging findings by two surgeons rather than intraoperative photographs. Although a unified database was designed using Excel software before data collection, this could still result in some bias. In addition, due to the selection bias caused by the surgeon preference, our study did not further analyze surgical options or prognosis of ML and OL among different surgeons, which might be the main factor influencing surgical approaches and methods. Furthermore, due to the lack of complete original images from the two centers, the imaging results of ML and OL in our study were not evaluated for preoperative distinction, which will be the focus of our future research.

## Conclusions

Our detailed analysis of ML and OL with the largest sample sizes from two centers suggested that compared with ML patients, OL patients are more prone to misdiagnosis and infection, but the prognosis of these patients after surgical treatment is better. Laparoscopic surgery could be a feasible surgical approach for treating OL patients.

## Data Availability

The datasets used and/or analysed during the current study are available from the corresponding author on reasonable request.

## Abbreviations

ML: Mesenteric lymphatic malformations
OL: Omental lymphatic malformations
